# Multi-organ gene expression analysis and network modeling reveal regulatory control cascades during the development of hypertension in female spontaneously hypertensive rat

**DOI:** 10.1371/journal.pone.0313252

**Published:** 2024-11-08

**Authors:** Eden Hornung, Sirisha Achanta, Alison Moss, James S. Schwaber, Rajanikanth Vadigepalli

**Affiliations:** Department of Pathology and Genomic Medicine, Daniel Baugh Institute for Functional Genomics and Computational Biology, Thomas Jefferson University, Philadelphia, Pennsylvania, United States of America; Juntendo University: Juntendo Daigaku, JAPAN

## Abstract

Hypertension is a multifactorial disease with stage-specific gene expression changes occurring in multiple organs over time. The temporal sequence and the extent of gene regulatory network changes occurring across organs during the development of hypertension remain unresolved. In this study, female spontaneously hypertensive (SHR) and normotensive Wistar Kyoto (WKY) rats were used to analyze expression patterns of 96 genes spanning inflammatory, metabolic, sympathetic, fibrotic, and renin-angiotensin (RAS) pathways in five organs, at five time points from the onset to established hypertension. We analyzed this multi-dimensional dataset containing ~15,000 data points and developed a data-driven dynamic network model that accounts for gene regulatory influences within and across visceral organs and multiple brainstem autonomic control regions. We integrated the data from female SHR and WKY with published multiorgan gene expression data from male SHR and WKY. In female SHR, catecholaminergic processes in the adrenal gland showed the earliest gene expression changes prior to inflammation-related gene expression changes in the kidney and liver. Hypertension pathogenesis in male SHR instead manifested early as catecholaminergic gene expression changes in brainstem and kidney, followed by an upregulation of inflammation-related genes in liver. RAS-related gene expression from the kidney-liver-lung axis was downregulated and intra-adrenal RAS was upregulated in female SHR, whereas the opposite pattern of gene regulation was observed in male SHR. We identified disease-specific and sex-specific differences in regulatory interactions within and across organs. The inferred multi-organ network model suggests a diminished influence of central autonomic neural circuits over multi-organ gene expression changes in female SHR. Our results point to the gene regulatory influence of the adrenal gland on spleen in female SHR, as compared to brainstem influence on kidney in male SHR. Our integrated molecular profiling and network modeling identified a stage-specific, sex-dependent, multi-organ cascade of gene regulation during the development of hypertension.

## Introduction

The hypertensive etiology involves dynamic dysregulation of systemic processes that evolve across multiple organ systems over time [1]. Multiple pathways contribute to the organ pathogenesis in hypertension including metabolic dysfunction in the liver [2, 3] and fibrotic dysregulation in the lungs [4], in addition to the well-recognized dysfunction of RAS [5, 6] in the kidney and adrenal gland. RAS is a physiological regulatory control system for sodium and blood pressure homeostasis, and the dysfunction of RAS has been suggested to be a mechanism contributing to the progression of hypertension [5]. Neurogenic hypertension arises from an imbalance in the activity of the sympathetic and parasympathetic nervous systems and resultant upward shift in the set point of blood pressure control [7]. Autonomic control of hypertension from the Central Nervous System (CNS) is maintained by sympathetic processes, which regulate the systemic renin-angiotensin system (RAS) and inflammation through the hypothalamic-pituitary-adrenal-axis (HPAA) [8]. In response to sympathetic processes, catecholamine secretion from the adrenal gland facilitates sodium resorption [9] and renin secretion in the kidney [10]. Sympathetic processes in the adrenal gland lead to increased cytokines and catecholamines, but the kidney has received much more attention as being the organ experiencing inflammation and end organ damage in hypertension [11]. The spleen has also been recently highlighted as potentially facilitating systemic inflammation and subsequent hypertension onset, in response to increased sympathetic processes [12]. Gene expression changes in multiple organs at physiologically relevant time points throughout the development of hypertension are likely to be indicative of the temporal cascade underlying the pathological processes of system-wide dysfunction.

Gene expression changes in the central autonomic circuits are expected to interact with and influence the pathway alterations occurring in peripheral organs including heart, adrenal gland, kidney, liver, lung, and spleen [13–18]. Our group recently described the regulatory interactions operating within and across organs in the male spontaneously hypertensive rat (SHR) during the development of hypertension and the corresponding age-matched normotensive genetic control Wistar-Kyoto rat (WKY) [13]. SHR is a widely used animal model to study the development of neurogenic hypertension [19–27]. SHR has been inbred to spontaneously develop hypertension over time, which enables the comparison of pre-hypertension, hypertension onset, and chronic hypertension stages. In male SHR, genes involved in inflammation, renin-angiotensin signaling, and sympathetic processes (*Il1b*, *Agt*, and adrenergic receptor subunits *Adra1b* and *Adrb2*) were differentially expressed across multiple organs and time points throughout the progression of hypertension [13]. Additionally, the brainstem network associated with male SHR exhibited a higher degree of connectivity in comparison to WKY control [13]. The organ-specific temporal cascade of gene activation and brainstem network connectivity relevant to these processes is relatively understudied in female SHR.

The present study is focused on characterizing the temporal cascade of differential gene expression during development of hypertension in female SHR. We integrated analysis of multi-pathway gene expression dynamics with a reverse engineering approach to infer data-driven dynamic network models of multi-organ gene regulatory influences. Through a combination of experimental and computational approaches, we interrogate temporal transcriptomic differences at the multi-organ scale and make predictions at both the organ-organ and gene-gene level, to infer shifts in the regulatory network topology during the development of hypertension. Our multi-pathway analysis identified organ-specific and system-wide gene expression changes during the onset and progression of hypertension.

## Results

**Fig 1:**
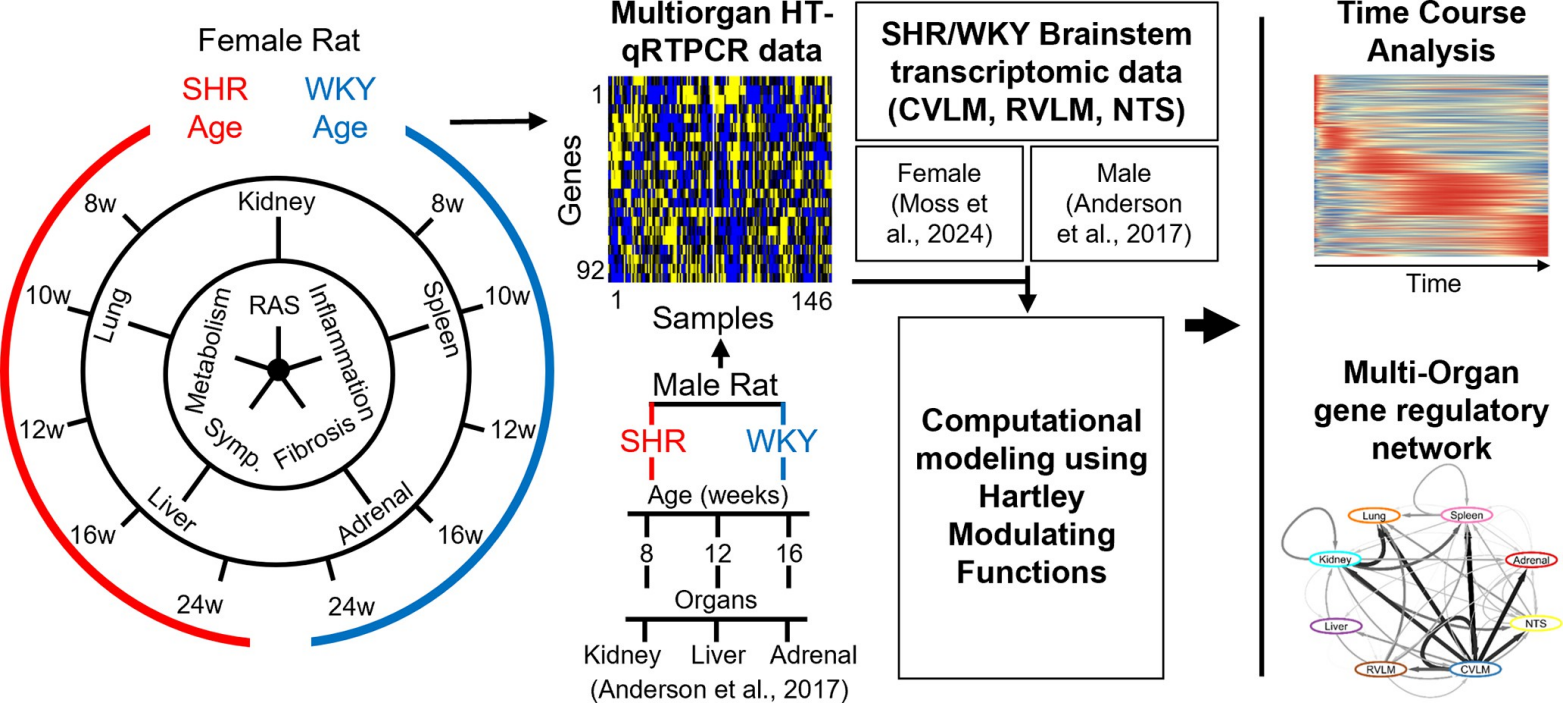
High throughput multi-organ multi-pathway time series gene expression data analysis and network modeling during the development of hypertension. A schematic of experimental design and computational workflow. 3 female SHR rats were compared to 3 age-matched female WKY rats at 8, 10, 12, 16, and 24 weeks of age during the development of hypertension. High throughput qRT-PCR data were obtained from the adrenal gland, kidney, liver, lung, and spleen for genes involved in sympathetic (Symp.) pathways, renin-angiotensin (RAS), inflammation, fibrosis, and metabolism. These data were contrasted with previously published male SHR data for the genes in common between the datasets [13]. Computational modeling using Harley Modulating Functions (HMF) to inform peak expression time course analysis and the generation of multi-organ gene regulatory networks incorporated previously published data from brainstem regions of the same female SHR animals [28].

We obtained multi-organ high-throughput qPCR gene expression data involved in inflammation, renin-angiotensin signaling, sympathetic pathways, metabolism, and fibrosis from adrenal gland, kidney, liver, lung, and spleen in female SHR (n = 3) and age-matched WKY control (n = 3) at time points corresponding to pre-hypertension (8 weeks), hypertension onset (10 and 12 weeks), and chronic hypertension (16 and 24 weeks) (Fig 1). This dataset represents an extension in scope of our previously published dataset from male SHR and WKY control including gene expression involved in inflammation, renin-angiotensin signaling, and sympathetic pathways from adrenal gland, kidney, and liver at time points corresponding to pre-hypertension (8 weeks), hypertension onset (12 weeks), and chronic hypertension (16 weeks) [13]. We subsequently performed data-driven multi-organ network modeling to infer gene regulatory influences in female SHR and WKY and compared the networks to our previously published findings in male SHR and WKY (2). Previously published time series gene expression data from brainstem regions from the same female SHR animals were incorporated for downstream analyses including computational modeling using Harley Modulating Functions (HMF) to inform peak expression time course analysis and the generation of multi-organ gene regulatory networks [28]. High-throughput qPCR data from female SHR and WKY were normalized within each respective organ. This dataset contained a total of 150 samples and 96 genes (14,400 data points). After quality control, a data matrix of 92 genes and 146 samples were considered in subsequent analysis (S1 Fig).

**Fig 2:**
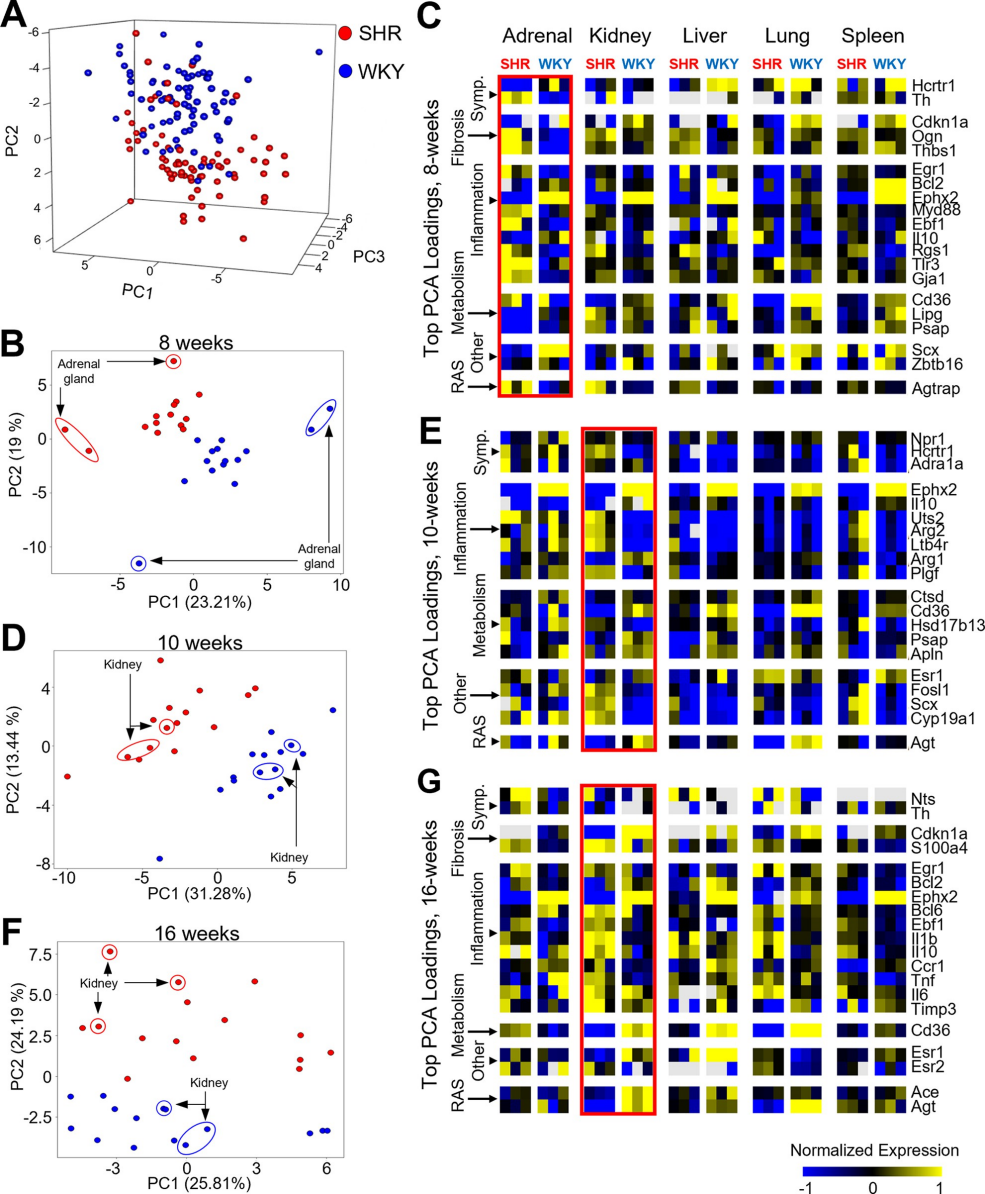
Stage-specific organ-specific drivers of transcriptomic variation across hypertension development in female SHR. A: Principal components (PCs) 1–3 for the female SHR and WKY multi-organ time series dataset containing 146 samples and 92 genes, colored for strain (Red: SHR, Blue: WKY). B: PC2 versus PC1 of the data subset for the 8-week time point, colored for strain, with the adrenal gland samples highlighted. C: Heat map of the top 20 genes positively and negatively contributing to PC2 at 8 weeks. D: PC2 versus PC1 of the data subset for the 10-week time point, colored for strain, with the kidney samples highlighted. E: Heat map of the top 20 genes positively and negatively contributing to PC1 at 10 weeks. F: PC2 versus PC1 of the data subset for the 16-week time point, colored for strain, with the kidney samples highlighted. G: Heat map of the top 20 genes positively and negatively contributing to PC2 at 16 weeks.

Principal component analysis (PCA) was used to visualize the dominant contributors to the variation in the transcriptomic data. Across all organs and time points, the data are largely separated by strain (SHR vs. WKY) along PC2 (Fig 2A). PCA of data from individual time points highlighted the greatest organ-specific variation across strains for each time point. This analysis suggested that major changes in gene expression occur in the adrenal gland at 8 weeks and the kidney at both 10 and 12 weeks (Fig 2B, 2D and 2F). The top 10 genes positively and negatively contributing to PC2 at 8 weeks highlight the strain-specific gene expression changes in the adrenal gland at 8 weeks, which include an upregulation of transcripts involved in catecholaminergic processes (*Th*), fibrosis (*Thbs1*), inflammation (*Tlr3*), and RAS (*Agtrap*) as well as a downregulation of other transcripts (such as the transcription factor *Scx*) in SHR (Fig 2C). Gene expression changes specific to the kidney occur later, at the hypertension onset (10 week) and robust hypertension (16 week) time points (Fig 2D–2G). At 10 weeks, transcripts involved in catecholaminergic processes (*Hctr1*), inflammation (*Ltb4r*), and other pathways such as hormone regulation (*Cyp19a1*) are upregulated in SHR, while transcripts involved in metabolism (*Cd36*) and RAS (*Agt*) are downregulated in SHR (Fig 2E). At 16 weeks, gene expression changes in the kidney involve an upregulation of transcripts involved in inflammation (*Il1b*, *Il10*) as well as downregulation of transcripts involved in metabolism (*Cd36*) and RAS (*Agt*) (Fig 2G). Taken together, these results point to the dynamics of differential gene expression that manifest across organs in distinct ways, with the earliest changes occurring in the adrenal gland followed by the kidney. Physiological implications include increased sympathetic activity from the adrenal gland via the HPAA being dynamically linked to the onset and development of hypertension in female SHR. Our results are also indicative of altered inflammatory gene regulation potentially leading to kidney dysfunction post-hypertension onset in female SHR.

### Organ-specific temporal cascade of gene expression

**Fig 3:**
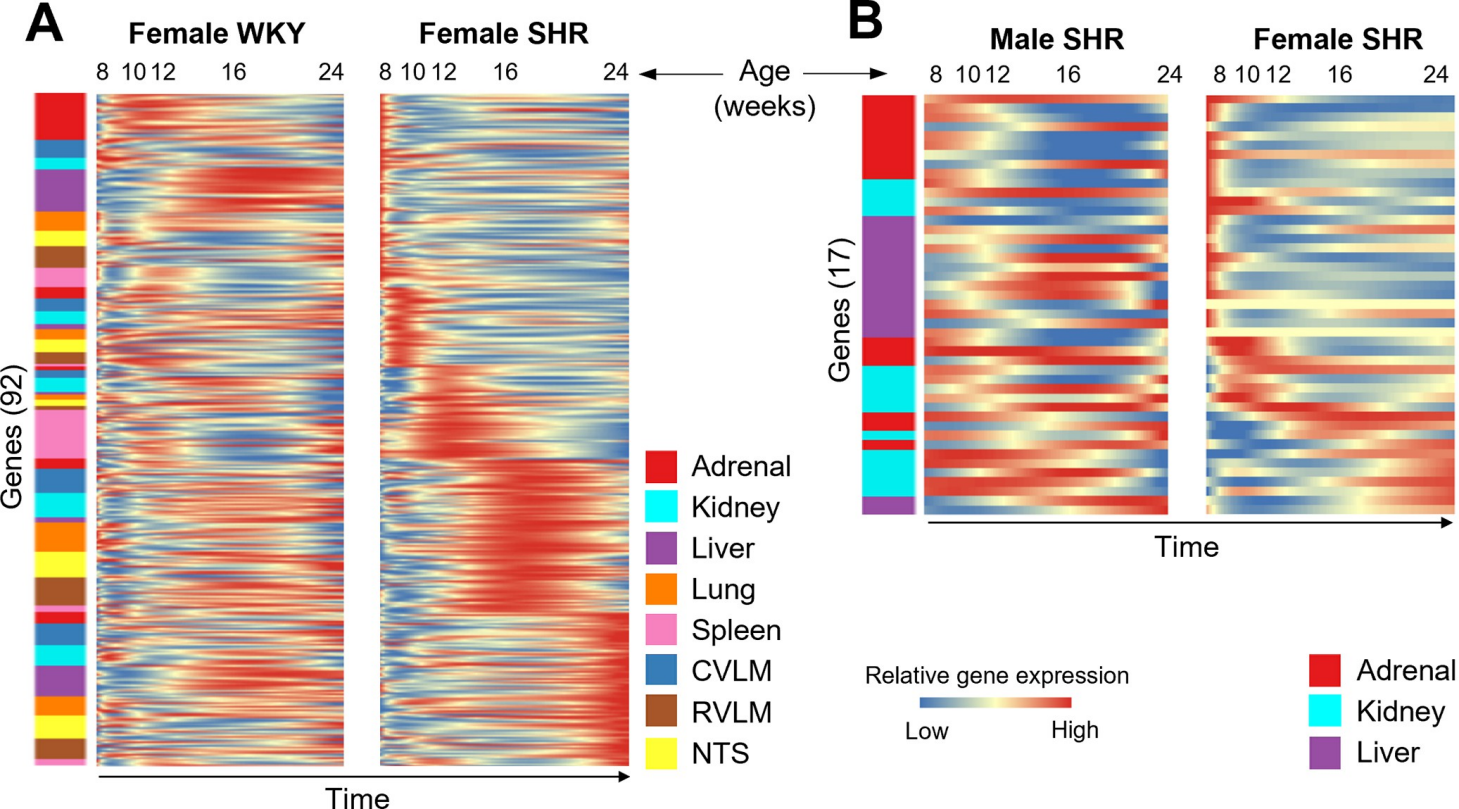
Model-predicted time course of multi-organ gene expression changes during the development of hypertension. The multi-organ gene regulatory network models for male and female SHR and WKY were simulated to predict the dynamic expression of the 92 genes across the inflammatory, RAS, metabolic, fibrotic and sympathetic pathways. The simulations were performed to span the ages from 8 to 24 weeks. A: Heat map of model-predicted expression levels of 92 genes across the 24-week time course in female SHR and WKY. The genes are ordered from top to bottom according to the time point of peak expression in SHR. B: Heat map of model-predicted expression levels of 17 genes in common between male and female datasets across the 24-week time course. The genes are ordered from top to bottom according to the time point of peak expression in female SHR.

To better understand potential gene regulatory networks governing multi-organ dynamics in hypertension, we employed a data-driven modeling approach. In addition to the data from the five peripheral organs, we incorporated previously published data from three brainstem regions (RVLM, CVLM, and NTS) of the same rats to include the influence of central autonomic circuits on multi-organ gene expression during hypertension progression [28]. We leveraged our discrete time series data from female SHR to impute a continuous-time model of gene expression dynamics using the Hartley Modulatory Function analysis method we have previously used to study multi-organ networks in male SHR [13]. The interaction coefficients in such a network model represent the inferred strength of the regulatory influence of genes on each other within and across organs. Modeling discrete time point data as continuous data enabled us to visualize model-predicted peak expression profiles across time, and ordering the imputed gene expression data revealed an autonomic dysfunction- specific cascade of gene activation across all 92 genes spanning inflammatory, RAS, metabolic, fibrotic and sympathetic pathways in female SHR distinct from that of WKY control (Fig 3A and S2A Fig).

Our analysis of female SHR and WKY data from five organs and three brain regions revealed a coordinated cascade of gene expression activation patterns across organs that are distinctly different between SHR and WKY. The cascade of gene activation in female SHR largely involves the adrenal gland and liver at 8 weeks as well as the spleen at 12 weeks of age (Fig 3A). In female WKY, however, the cascade of gene activation largely involves the lung and spleen at 8 weeks, the adrenal gland at 12 weeks, the liver and kidney at 16 weeks, and the NTS at 24 weeks of age (S2A Fig). These findings suggest that, in female SHR, gene expression peaks emerge much earlier in the adrenal gland and liver, as well as much later in the spleen as compared to female WKY. We also observed a diminished degree of gene activation in the lung at 8 weeks as well as in the kidney at 16 weeks in female SHR as compared to female WKY. Organ-specific sex differences are evident early, as several brainstem gene expression profiles showed peak upregulation at the pre-hypertensive age of 8 weeks in males [13], while female-specific peak gene expression is first observed in the adrenal gland and liver at 8 weeks (Fig 3A). These results point to the physiological role of the brainstem in governing hypertension onset in male SHR, as opposed to the adrenal gland and liver initiating hypertension onset in female SHR. Diminished regulatory control from the spleen and lungs at the pre-hypertension stage is suggestive of a loss of function mechanism with physiological implications leading to hypertension onset in female SHR vs WKY.

To identify sex-specific cascades of gene expression activation amongst SHR profiles, we interrogated male SHR vs. female SHR gene expression activation patterns across the 17 genes and three organs in common between the different datasets. Early peaks were similarly observed for adrenal gland in both female and male SHR (Fig 3B and S2B Fig). In female SHR, however, gene activation in the liver was the most dominant at 8 weeks, while instead the adrenal gland and kidney both show dominant gene expression activation peaks in male SHR at 8 weeks (Fig 3B and S2B Fig). Early peaks were observed for kidney *Tgfb1* in pre-hypertensive males at 8 weeks [13], while kidney-specific gene expression peaks emerged much later in females (S2B Fig). This analysis suggests gene upregulation in the liver of pre-hypertensive female SHR and gene upregulation in the kidney of pre-hypertensive male SHR correspond to the organ-specific contributions to sex differences in the pathogenesis of hypertension. Interestingly, however, the sex-specific gene expression changes in multiple organs appear to be opposite at hypertension onset and chronic hypertension, where we see gene upregulation in the kidney in female SHR and in the liver in male SHR (Fig 3B and S2B Fig). These results indicate that the pathogenesis of female hypertension begins in the adrenal gland and liver, but later manifests in the kidney, while the pathogenesis of male hypertension begins in the adrenal gland and kidney, but later manifests in the liver. Depending on sex, organ-specific alterations may be differentially diagnostic of disease progression.

### Modeling divergent SHR-specific regulatory networks

**Fig 4:**
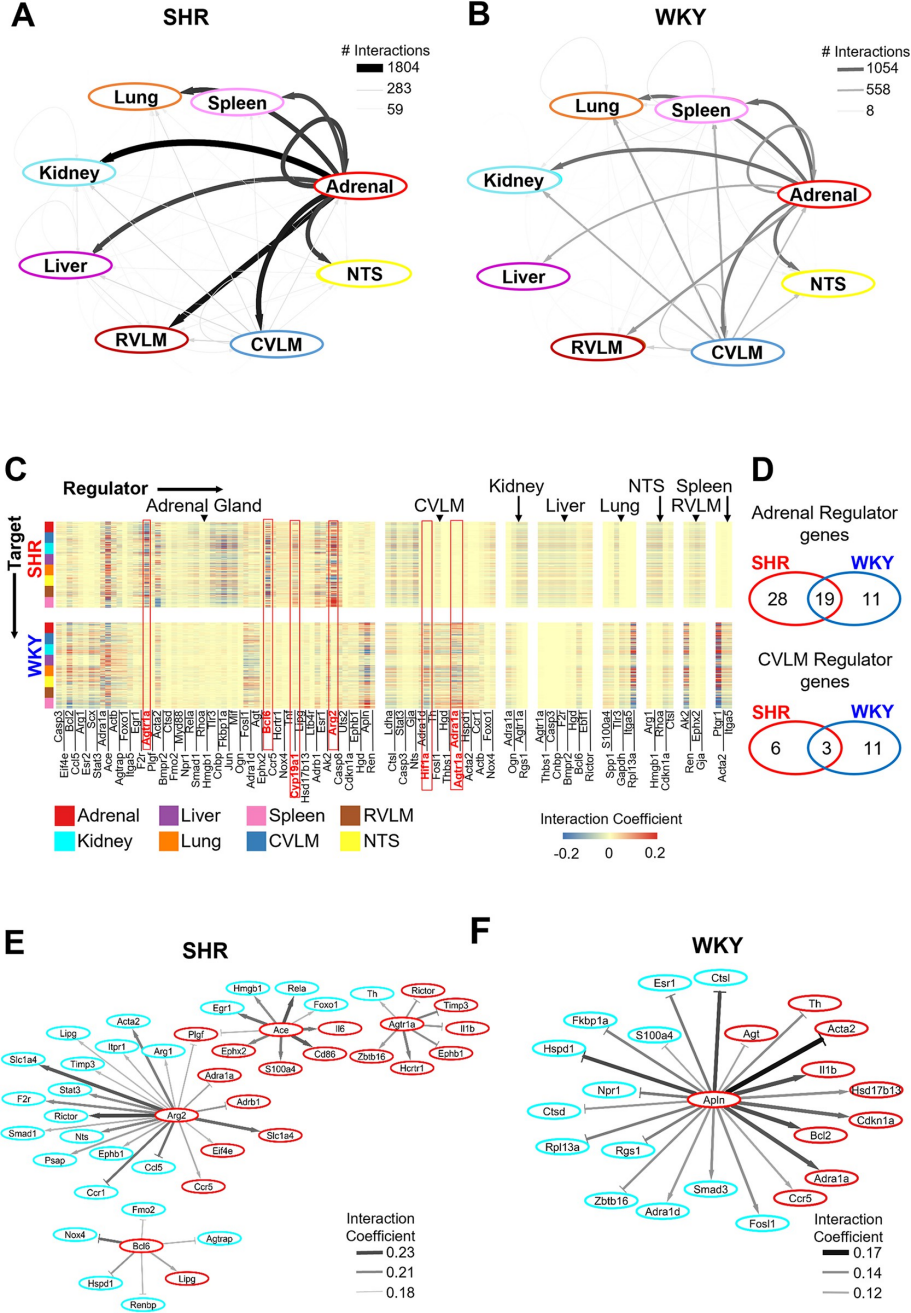
Multi-organ gene regulatory networks in female SHR vs. WKY. A,B: Network of organ-organ interactions in SHR (panel A) and WKY (panel B). The edge thickness and color are mapped to the number of interaction coefficients that are at least two standard deviations from the mean, as a heuristic threshold for illustrative purposes. C: Heat map of regulatory matrix containing the interaction coefficients that are at least two standard deviations from the mean in female SHR (top) and WKY (bottom). The columns of the matrix correspond to the regulatory genes in the network in any given organ. The rows correspond to the target genes within and across organs. Positive interaction coefficients are colored red in the matrix, while negative interaction coefficients are colored blue. The highlighted columns represent selected key regulators in the network (described in the Results section). D: Total number of times each organ, adrenal gland (top) and CVLM (bottom), shows up as a regulator in panel C in SHR (left), WKY (right) or both (middle). E,F: Network of gene-gene interaction coefficients in SHR (panel E) and WKY (panel F) across adrenal gland and kidney. The edges shown correspond to the absolute interaction coefficients greater than 75% of maximum value (SHR) and 25% of maximum value (WKY). The edge thickness and color are mapped to the level of the interaction coefficients.

We developed network models of gene regulatory influence within and across organs based on the time series gene expression data in male and female SHR and WKY. While both time series from male and female datasets consist of five time points, only three time points are in common between the two datasets. By placing both datasets in the context of an overarching network model we can more easily compare the sex-dependent changes between them. We compared the network models to gain insight into model-predicted organ-organ and gene-gene regulatory interaction strengths in female SHR vs. WKY. Analysis of the network structures suggests that the regulatory interactions from the adrenal gland, and to a much lesser extent the liver, are strengthened in the autonomic dysfunction phenotype of SHR as compared to WKY control (Fig 4A–4D). More specifically, at the gene level, *Agtr1a*, *Bcl6*, *Cyp19a1*, and *Arg2* from the adrenal gland are predicted by the model to have an increased interaction strength in female SHR as compared to WKY (Fig 4C). Regulatory interactions from the caudal ventrolateral medulla (CVLM), however, are diminished in the autonomic dysfunction phenotype as compared to control (Fig 4A–4D). The network model indicates that *Hif1a*, *Adra1a*, and *Agtr1a* from the CVLM are likely to have a decreased influence in female SHR as compared to WKY (Fig 4C). The inferred influence of *Agtr1a* from the CVLM is diminished, while *Agtr1a* from the adrenal gland is strengthened in female SHR. 47 genes were predicted to exert regulatory control from the adrenal gland in female SHR as compared to only 30 genes in female WKY (Fig 4D, top). There was an overlap of 19 genes between the two networks, leaving 28 SHR-specific regulatory genes and only 11 WKY-specific regulatory genes from the adrenal gland (Fig 4D, top). In the CVLM, only 9 genes were predicted to be influential in female SHR, and of these 9 genes, 3 were in common with the WKY network, yielding 6 SHR-specific regulatory genes as well as 11 WKY-specific regulatory genes in the CVLM (Fig 4D, bottom). These findings highlight the potential role of increased gene regulatory influence from the adrenal gland in female SHR as well as the potential diminished role of gene regulatory influence from the CVLM in female SHR. Collectively, this analysis suggests a highly influential role of the multi-pathway gene expression changes in adrenal gland as well as an attenuation of gene regulatory influence from the autonomic control circuit in the female hypertensive etiology.

Given the apparent early gene expression activation in the adrenal gland and subsequent gene expression activation in the kidney in female SHRs (Fig 3), we sought to interrogate the possible processes involved in hypertension progression from the adrenal gland to the kidney in female SHRs. We extracted the top 75% strongest gene-gene interaction coefficients predicted by the model across these two organs in female SHR (Fig 4E, middle). The majority of genes in the top 75% were from adrenal gland, rather than kidney (Fig 4E, middle). We then extracted the top 25% gene-gene interaction coefficients across adrenal gland and kidney in female SHR (Fig 4E, left). From this analysis we have identified that adrenal *Bcl6*, *Arg2*, *Ace*, and *Agtr1a* are predicted to have a strong regulatory influence over several other genes in both the adrenal gland as well as the kidney in female SHR (Fig 4E, left). The top 75% strongest gene-gene interaction coefficients from female WKY adrenal gland and kidney suggests that *Apln* from the adrenal gland is predicted to have a strong regulatory influence over several other genes in both the adrenal gland and the kidney (Fig 4E, right) Taken together, the results from this analysis suggest that regulatory control from the adrenal gland, over the adrenal gland and kidney, shifts from *Apln* in female WKY to *Bcl6*, *Arg2*, *Ace*, and *Agtr1a* in the female SHR. This suggests that processes involving inflammation (*Bcl6*), oxidative stress (*Arg2*), and RAS (*Ace* and *Agtr1a*) in the adrenal gland may out compete the regulatory role of *Apln* over several processes in both the adrenal gland and kidney of female SHR.

**Fig 5:**
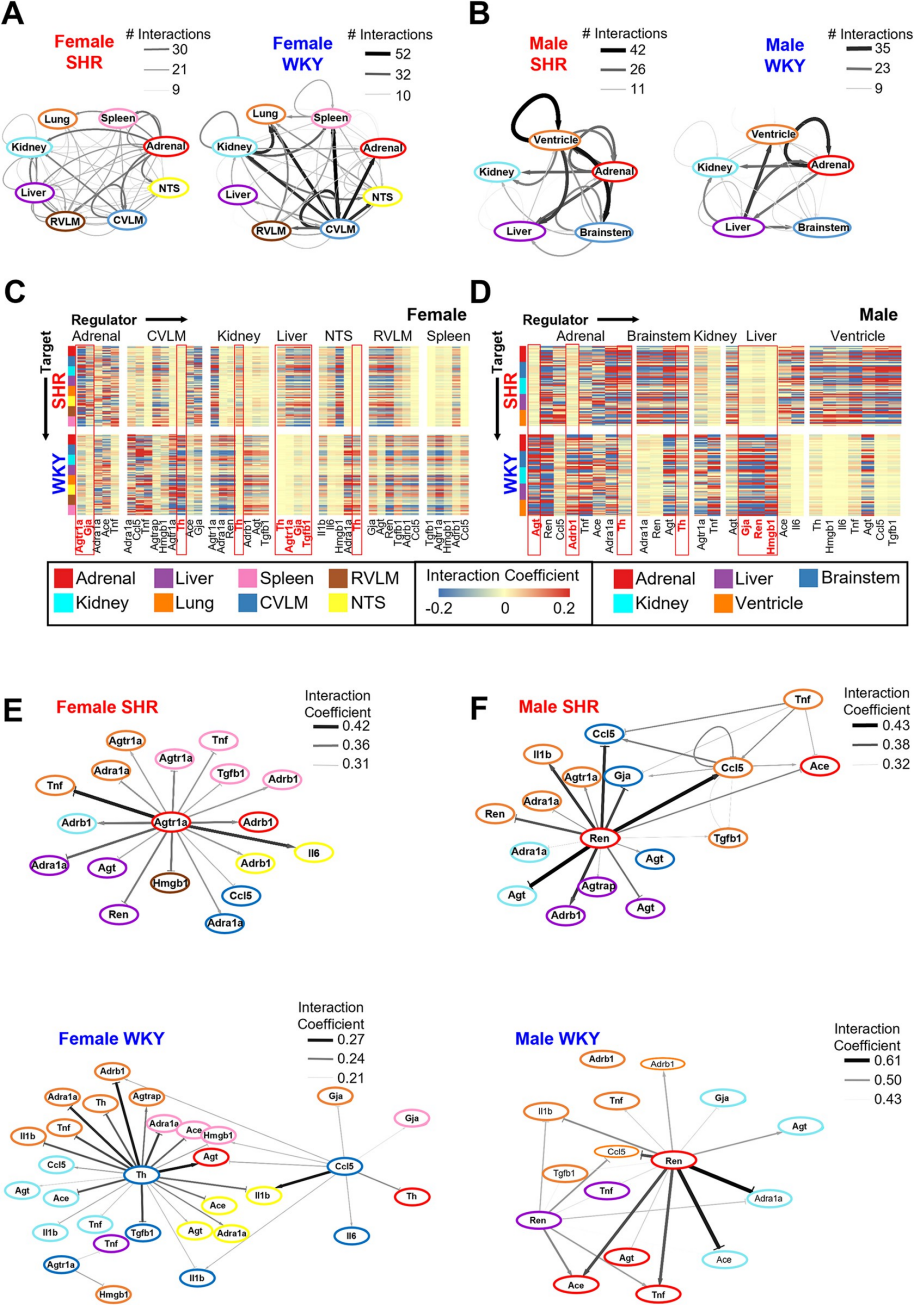
Comparison of multi-organ gene regulatory networks in male and female SHR vs. **WKY.** A,B: Network of organ-organ interactions in SHR (left) and WKY (right) (A: female, B: male). The edge thickness and color are mapped to the number of interactions within two standard deviations of the mean. C-D: Heat map of interaction coefficients within two standard deviations of the mean in SHR (top) and WKY (bottom) (C: female, D: male). E,F: Network of organ-organ interactions in SHR (top) and WKY (bottom) in female (panel E) and male (panel F). The edges shown correspond to the absolute interaction coefficients greater than 75% of maximum value (female SHR) and 25% of maximum value (female WKY, as well as male SHR and WKY). The edge thickness and color are mapped to the level of the interaction coefficients.

We next wanted to compare male and female gene-gene and organ-organ interaction networks for the 17 genes in common between the datasets. From this smaller network, the model similarly predicted an increased number of interactions from adrenal gland and liver, as well as a decreased number of interactions from CVLM, in female SHR vs. WKY control (Fig 5A). In male SHR, however, we observed the opposite. The model predicted a decreased number of interactions from the adrenal gland and liver, but an increased number of interactions from the brainstem in male SHR vs. WKY (Fig 5B) [13]. At the gene level, the model inferred decreased regulatory control from *Th* in both the CVLM and the NTS in female SHR as compared to control (Fig 5C). However, the model-predicted an increased role of *Th* from the brainstem, as well as the adrenal gland, in male SHR as compared to control (Fig 5D). In female SHR, *Th* from the kidney as well as liver were predicted by the model to have stronger interaction coefficients as compared to WKY control (Fig 5C). The model also inferred an increased regulatory influence of *Agtr1a* and *Gja* from adrenal gland in female SHR vs. WKY (Fig 5C). Other transcripts in the liver predicted to have stronger interaction coefficients in female SHR vs. WKY include *Agtr1a*, *Gja*, and *Tgfb1* (Fig 5C). In contrast to the upregulated influential control predicted to be from adrenal gland and liver in female SHR, *Agt* and *Adrb1* from the adrenal gland, as well as *Gja*, *Ren*, and *Hmgb1* from the liver, are predicted to have weakened regulatory control in male SHR vs. WKY control (Fig 5D). These findings suggest that individual organs exert differential regulatory control in distinct sex-dependent ways to manifest the pathogenesis of hypertension. More specifically, our analyses suggest that the adrenal gland and liver may exert increased gene regulatory control leading to the development of hypertension in females, while the influence of gene regulatory control from adrenal gland and liver may instead be attenuated during the development of hypertension in males. Our results also suggest that the upregulated gene regulatory influence of the brainstem in hypertension development is male-specific.

The smaller model-predicted network of gene-gene and organ-organ interactions across the 17 genes in common with the male dataset was subset further for the top 25% of interaction coefficients (Fig 5E). This network suggests strong gene regulatory control from adrenal *Agtr1a* over several transcripts in all tissue types studied in female SHR (Fig 5E). The female WKY data were subset for the top 75% strongest inferred interaction coefficients (Fig 5E, bottom). This network highlights the strong gene regulatory influence of *Th*, *Ccl5*, *Agtr1a*, and *Il1b* from the CLVM in female WKY (Fig 5E, bottom). Taken together, these analyses support an enhanced gene regulatory influence of adrenal *Agtr1a* as well as a diminished gene regulatory influence of *Th*, *Ccl5*, *Agtr1a*, and *Il1b* from the CVLM in regulating the progression of hypertension in females. The model-predicted network from male SHR data were also subset for the top 75% highest interaction coefficients (Fig 5F, top). This network highlights the prominent gene regulatory influence of adrenal *Ren* over several transcripts in all tissues studied from male SHR (Fig 5F, top). The top 75% highest interaction coefficients in male WKY also included adrenal *Ren* (Fig 5F, bottom). However, in male WKY, adrenal *Ren* is not predicted by the model to have strong regulatory control over any transcripts in the brainstem or liver (Fig 5F, bottom). Taken together, this work indicates that *Ren* from the adrenal gland may dysregulate transcriptomic networks in the brainstem and liver in male SHR. This analysis suggests that transcriptomic networks of the adrenal gland as well as the brainstem are dysregulated in the development of hypertension in sex-dependent ways.

### Multi-organ differential gene expression dynamics

#### Catecholaminergic processes

**Fig 6:**
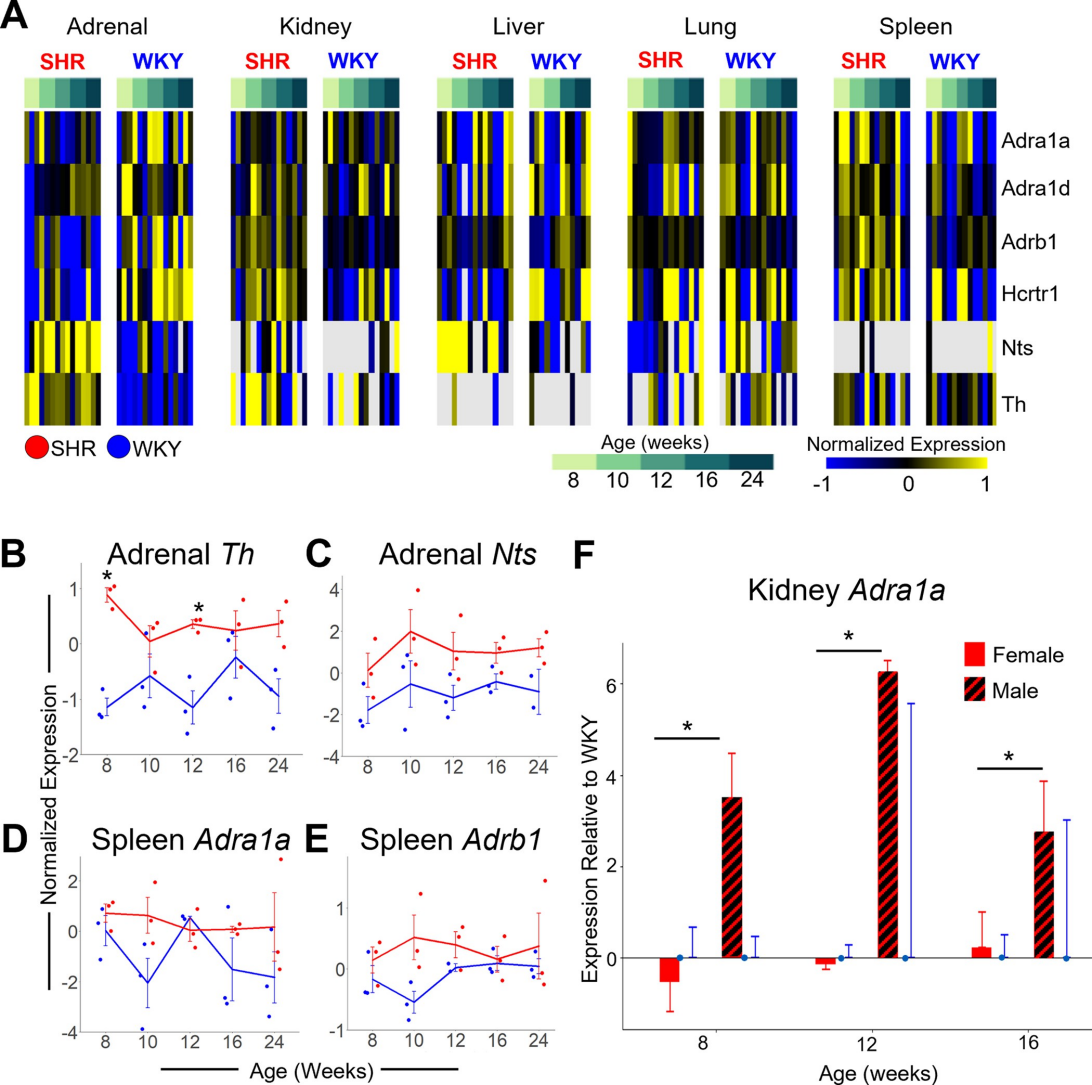
Multi-organ gene expression changes in catecholaminergic processes across the development of hypertension. A: Heat map of catecholaminergic-related gene expression across 146 samples in the female SHR and WKY dataset. B-E: Relative gene expression (- ΔΔCt) of *Th* (panel B) and *Nts* (panel C) (*strain p = 0.001) in adrenal gland as well as *Adra1a* (panel D) (strain p = 0.0508) and *Adrb1* (panel E) (strain p = 0.02) in spleen in SHR (red) and WKY (blue). *Th* is significantly enriched in SHR adrenal gland at 8 (p = 0.002) and 12 weeks (p = 4 x 10^−6^) as compared to WKY. F: Differential expression of *Adra1a* in the SHR kidney relative to WKY in female (solid) and male (striped). *Adra1a* is significantly upregulated in male SHR kidney compared to female at 8 (p = 1 x 10^−3^), 12 (2 x 10^−6^), and 16 (0.01) weeks. Error Bars represent +/- standard error of the mean. * p < = 0.01.

We assayed for several transcripts involved in the activation (*Hctr1*), synthesis (*Th*, *Nts*), and downstream signaling (*Adra1a*, *Abrb1*) of catecholamines to gain a deeper understanding of the sex-specific organ differences of sympathetic pathways in the development of hypertension (Fig 6A). *Hcrtr1* is downregulated across all time points studied in female SHR vs. WKY in the adrenal gland (p = 3 x 10^−4^; Fig 6A). *Hcrtr1* activates glucocorticoid production [29], which in turn negatively regulates adrenocorticotropin-releasing hormone (ACTH) secretion from the pituitary gland and thereby negatively regulates catecholamine secretion [30]. Downregulation of *Hcrtr1* suggests catecholamine secretion from the adrenal gland is likely to be upregulated in female SHR. Expression of *Th* (p = 1.76 x 10^−6^), the rate-limiting enzyme in catecholamine synthesis, and *Nts* (p = 1 x 10^−3^, age-independent), which induces *Th* expression [31], are both significantly upregulated in the adrenal gland of female SHR vs. WKY control (Fig 6B and 6C). Catecholamines released from the adrenal gland activate adrenergic receptors at the spleen to stimulate the systemic immune response [32]. Adrenergic receptors, *Adra1a* (p = 0.05) and *Adrb1* (p = 0.02), are both significantly upregulated in the spleen of female SHRs as compared to WKY (Fig 6D and 6E). These findings suggest that autonomic dysfunction manifests with an increase in catecholaminergic transmission from the adrenal gland to the spleen, and physiological implications including subsequently increased systemic inflammation, in female SHR. Circulating catecholamines activate the adrenergic receptor ADRA1A in the kidney and have a direct influence on vasoconstriction and renin release leading to sodium reabsorption [33]. *Adra1a* is significantly upregulated in the kidney of male SHR vs. WKY (Fig 6F). This finding paired with our previous report of increased gene regulatory influences from the brainstem in male SHR vs. WKY suggests autonomic dysfunction manifests with an increased catecholaminergic transmission from the brainstem to the kidney, and physiological implications including vasoconstriction and renin release leading to sodium reabsorption, in male SHR [13]. Taken together, these results suggest sex-dependent differences in catecholaminergic processes throughout the progression of hypertension, highlighting the role of cell signaling from the adrenal gland to the spleen in female SHR, as well as from the brainstem to the kidney in male SHR.

## Renin-angiotensin system

**Fig 7:**
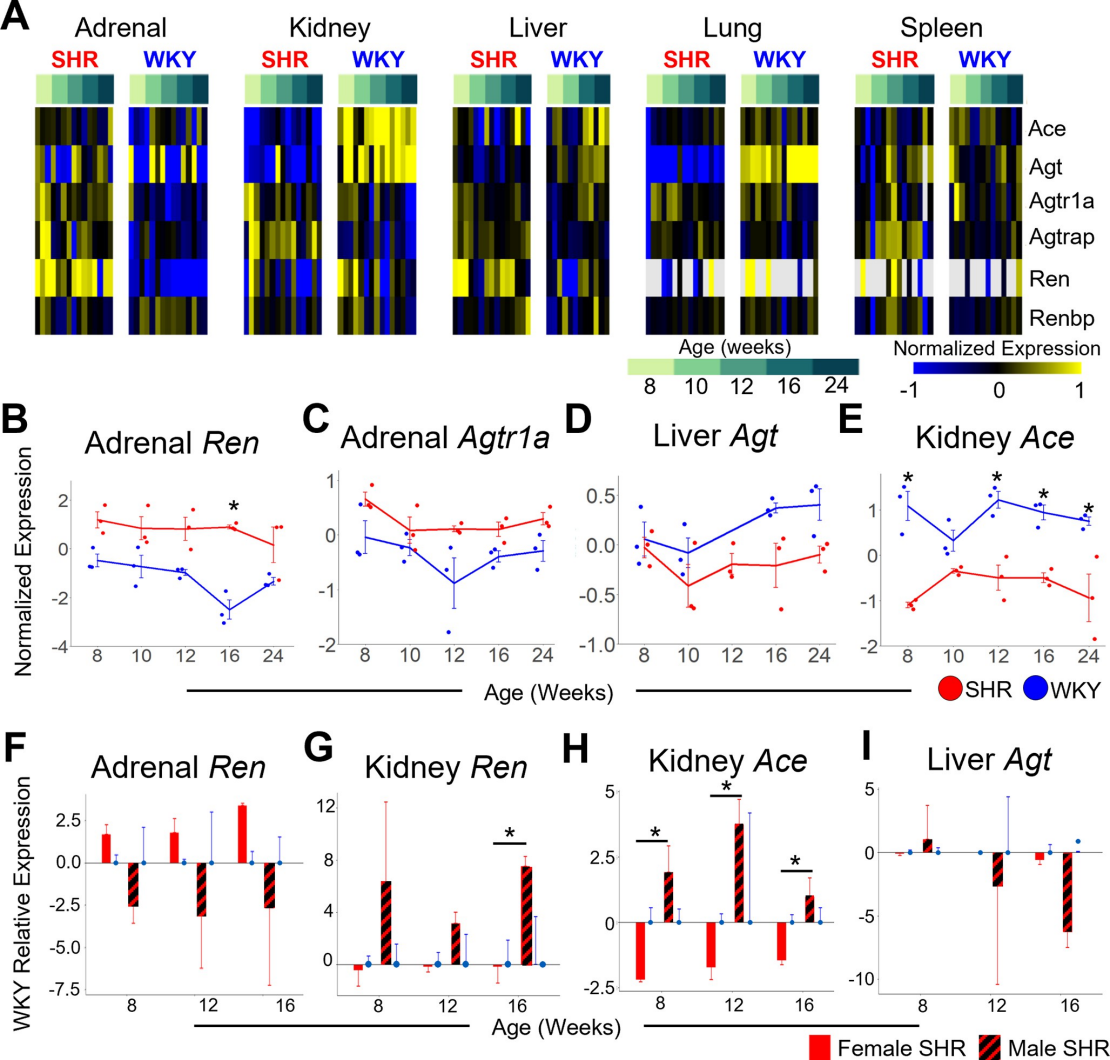
Multi-organ gene expression dynamics related to the Renin-Angiotensin System across the development of hypertension. A: Heat map of RAS-related gene expression across 146 female samples. B-E: Relative expression (–ΔΔCt) of *Ren* (panel B) and *Agtr1a* (panel C) in adrenal gland as well as *Agt* in liver (panel D), and *Ace* in kidney (panel E), annotated for strain (SHR: red, WKY: blue). Adrenal *Ren* is significantly higher in SHR at 16 weeks compared to WKY (p = 0.03). *Agtr1a* is significantly higher in SHR adrenal gland compared to WKY (strain p = 2 x 10^−4^). *Ag*t is significantly higher in SHR liver compared to WKY (strain p = 0.004). *Ace* is significantly lower in SHR kidney compared to WKY at 8 (p = 1 x 10^−4^), 12 (p = 0.02), 16 (p = 0.01), and 24 week (p = 0.003). F-I: *Ren* expression in SHR adrenal gland (panel F) (sex p = 4 x 10^−4^) and kidney (panel G), as well as *Ace* expression in SHR kidney (panel H) and *Agt* expression in SHR liver (panel I), all relative to WKY in female (solid) and male (striped). *Ren* is significantly upregulated in female SHR compared to male SHR adrenal gland (sex p = 4 x 10^−4^) and *Ren* is significantly upregulated in male SHR kidney as compared to female at 16 weeks (p = 0.04). *Ace* is significantly more upregulated in male SHR than female SHR kidney (8 weeks p = 3 x 10^−5^, 12 weeks p = 2 x 10^−6^, 16 weeks p = 0.006). Error Bars represent +/- standard error of the mean.

To investigate the sex-specific organ differences of components of the peripheral renin-angiotensin system (RAS), we assayed for several transcripts involved in RAS, including *Ace*, *Agt*, *Agtr1a*, *Agtrap*, *Ren*, and *Renbp* (Fig 7A). Circulating renin (REN) is produced by the kidneys in response to renal artery hypotension, decreased Na^+^ load delivery to the distal tubule in the kidney, and sympathetic activation in response to reduced arterial blood pressure [34–36]. Circulating REN converts angiotensinogen (AGT), primarily produced in the liver, to angiotensin I [37]. Angiotensin I is then converted to angiotensin II by angiotensin converting enzyme (ACE), primarily found in the vascular endothelium of the lungs and kidneys [37]. AGTR1A is the receptor for angiotensin ll, and AGTRAP is involved in the internalization of AGTR1A after angiotensin ll activation, leading to subsequent aldosterone activation [38].

Sex-dependent differential expression of RAS-related genes was observed across all organs studied (Fig 7A). Renin mRNA (*Ren*) was not differentially expressed in the kidney of female SHR at any time point compared to WKY (Fig 7A). However, intra-adrenal RAS components, including *Ren* (p = 1.36 x 10^−7^) and *Agtr1a* (p = 2 x 10^−4^), were upregulated in the adrenal gland of female SHR vs. WKY (Fig 7B–7C). This enrichment of RAS-related gene expression in the adrenal gland occurs early during the development of hypertension and is sustained throughout chronic hypertension (Fig 7B–7C). In contrast to intra-adrenal RAS components, endogenous sources of systemic circulatory RAS, including liver *Agt* (p = 4 x 10^−3^) and kidney *Ace* (p = 3.27 x 10^−9^), were downregulated in female SHR vs. WKY control (Fig 7D–7E). *Ren* was downregulated in the adrenal gland of male SHR vs. WKY control (Fig 7F), while endogenous systemic circulatory RAS components canonically produced by the kidney (*Ren* and *Ace*) were upregulated in the kidney of male SHR vs. WKY control (Fig 7G–7H). These findings highlight the sex-dependent and organ-specific differences in RAS gene expression dynamics in SHR. The endogenous systemic circulatory RAS component canonically produced by the liver (*Agt*) was downregulated in SHR across both sexes as compared to WKY control (Fig 7I). Overall, our analyses suggest that augmented expression of intra-adrenal RAS-related genes is a sex-specific etiology of autonomic dysfunction in female SHR, while enhanced expression of endogenous systemic circulatory sources of RAS-related transcripts in the kidney is a sex-specific etiology of autonomic dysfunction in male SHR. Plasma renin levels, presumably from the kidney, have been previously reported to be higher in males than females [39]. Together, these results suggest that the main source of the renin-angiotensin system may have shifted away from the kidney-liver-lung axis to the adrenal gland in female SHR.

## Immune system

**Fig 8:**
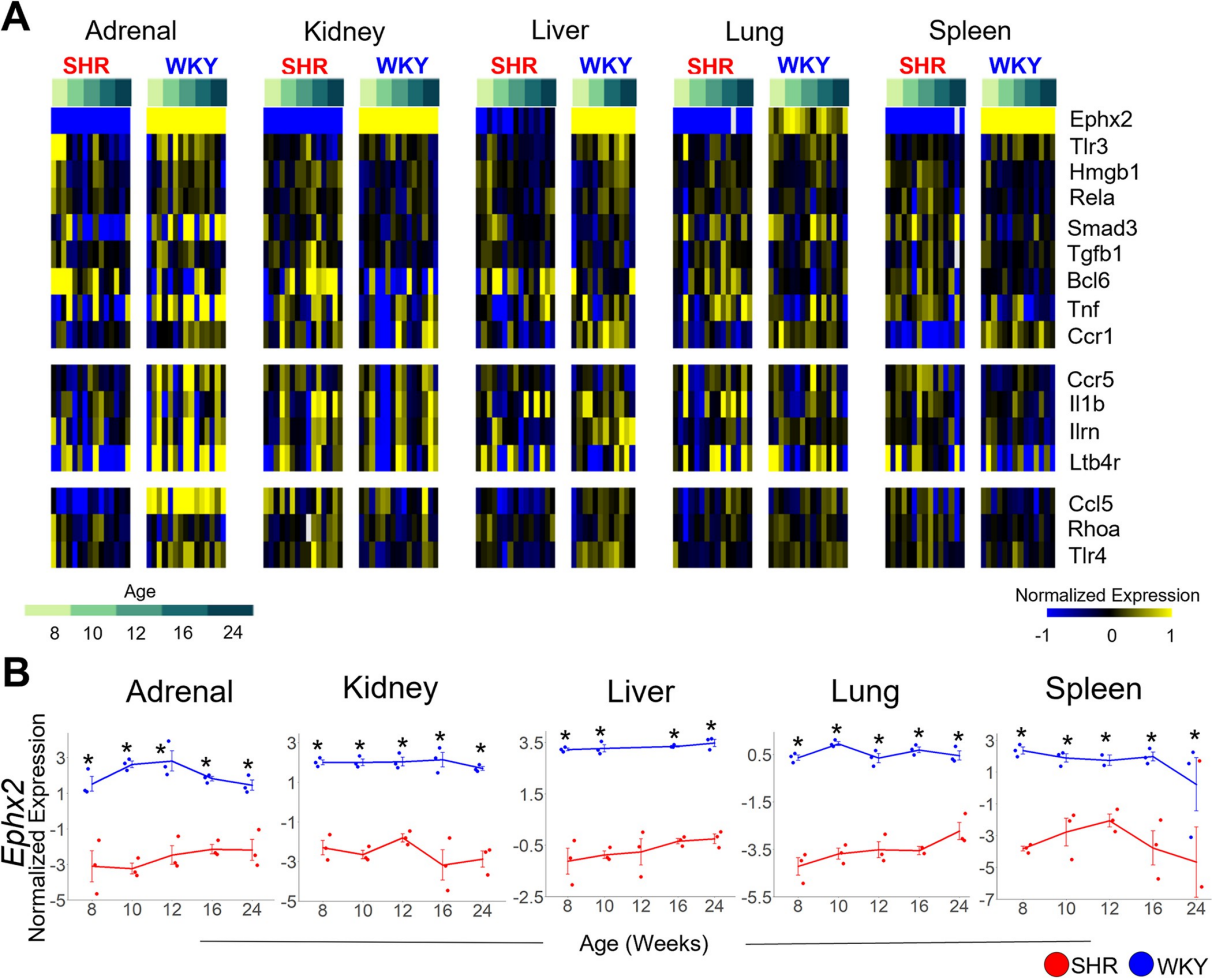
Multi-organ gene expression dynamics related to inflammatory processes in female SHR. A: Heat map of inflammation-related gene expression across 146 samples in female SHR and WKY. B: Relative gene expression (–ΔΔCt) of *Ephx2* across 5 organs, annotated for strain (SHR: red, WKY: blue). Error Bars represent +/- standard error of the mean. (*p < 0.05, ANOVA Tukey HSD post-hoc p-value).

To assess the immune profile in the pathogenesis of female hypertension, we performed a chronological assessment of gene expression levels of various cytokines and their cognate receptors (Fig 8A). Inflammatory markers, including *Tlr3* and *Bcl6*, are upregulated in the adrenal gland early on during pre-hypertension at 8 weeks in female SHR vs. WKY, while other inflammatory markers, including *Tnf*, *Il1b*, *and Ltb4r*, are upregulated in the kidney at later time points including hypertension onset at 10 weeks and established hypertension at 16 weeks in female SHR vs. WKY control (Fig 8A). The overall trend of increased expression of inflammatory markers across all organs studied is paired with a seemingly compensatory systemic downregulation of *Ephx2* (only assayed for in females) across multiple organs and time points in female SHR as compared to WKY control (Fig 8A–8B).

**Fig 9:**
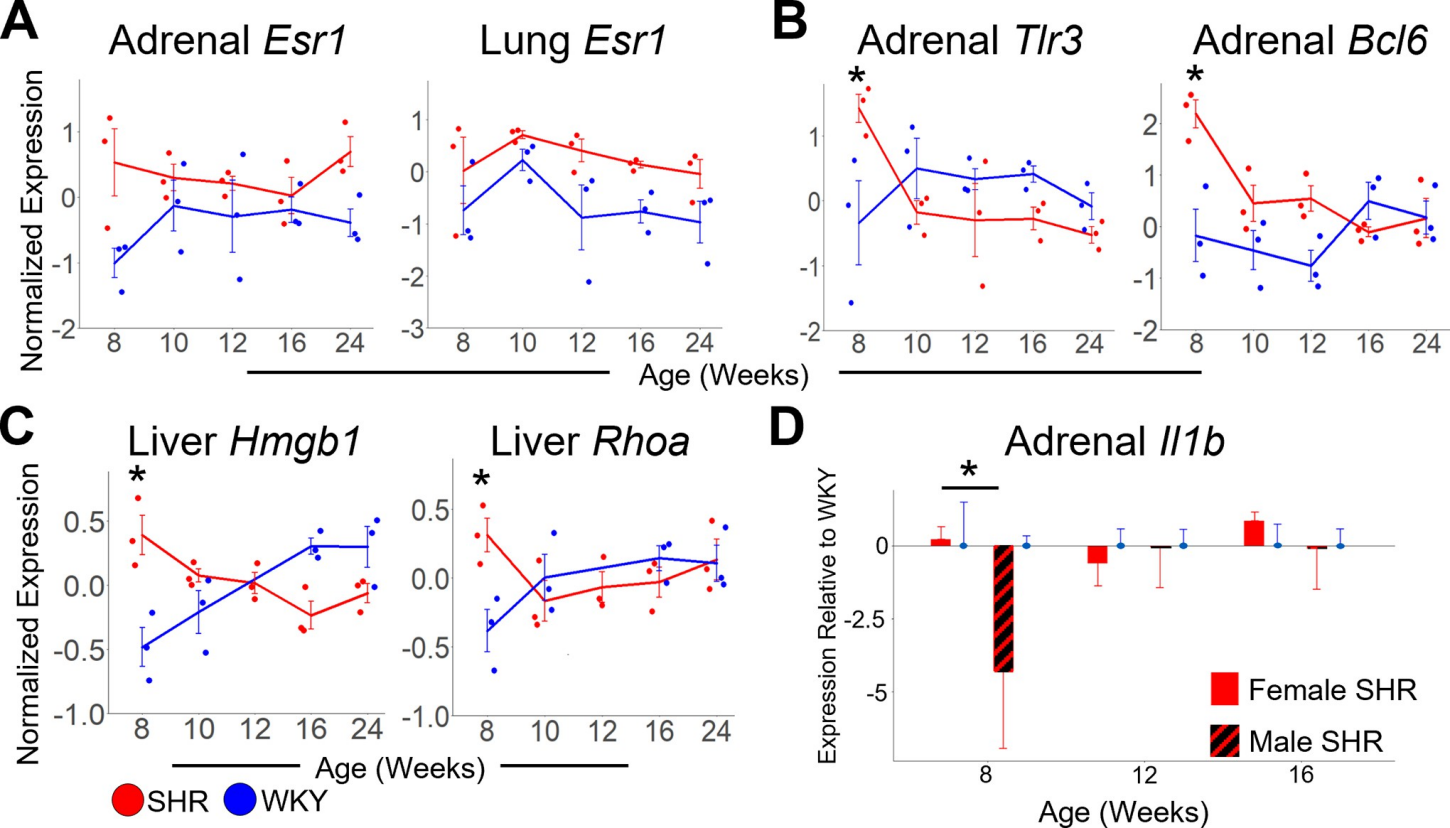
Multi-organ differential gene expression statistically significant at the pre-hypertensive stage. A: Relative expression (–ΔΔCt) of *Esr1* in adrenal gland (left) and lung (right), annotated for strain (SHR: red, WKY: blue). *Esr1* is significantly upregulated in SHR adrenal gland compared to WKY (strain p = 0.002) and in SHR lung compared to WKY (strain p = 0.001). B: Relative expression (–ΔΔCt) of *Tlr3* (left) and *Bcl6* (right) in adrenal gland. Adrenal *Tlr3* is significantly higher in SHR at 8 weeks as compared to WKY (p = 0.04). Adrenal *Bcl6* is significantly higher in SHR at 8 weeks compared to WKY (p = 0.002). C: Relative expression (–ΔΔCt) of *Hmgb1* (left) and *Rhoa* (right) in liver. *Hmgb1* is significantly higher in SHR liver at 8 weeks as compared to WKY (p = 0.002). *Rhoa* is significantly higher in SHR liver at 8 weeks as compared to WKY (p = 0.04). D: Adrenal *Il1b* expression relative to WKY in female (solid) and male (striped) SHR. *Il1b* is significantly downregulated in male SHR adrenal gland compared to female at 8 weeks (p = 0.02). Error Bars represent +/- standard error of the mean. All p-values indicated are from a post-hoc Tukey Honest Significance test.

Downregulation of EPHX2 has been attributed to estrogen in females [40] and the estrogen receptor, ESR1, inhibits pro-inflammatory cell expansion [41]. Therefore, we sought to elucidate whether *Esr1* was differentially expressed amongst female SHR and WKY organs. *Esr1* is significantly upregulated in female SHR adrenal gland (strain p = 0.002; Fig 9A, left) and lung (strain p = 0.001; Fig 9A, right), but downregulated in female SHR spleen (strain p = 0.005) as compared to normotensive control. This supports a potential anti-inflammatory role for *Esr1* in female SHR adrenal gland and lung, but not spleen.

**Fig 10:**
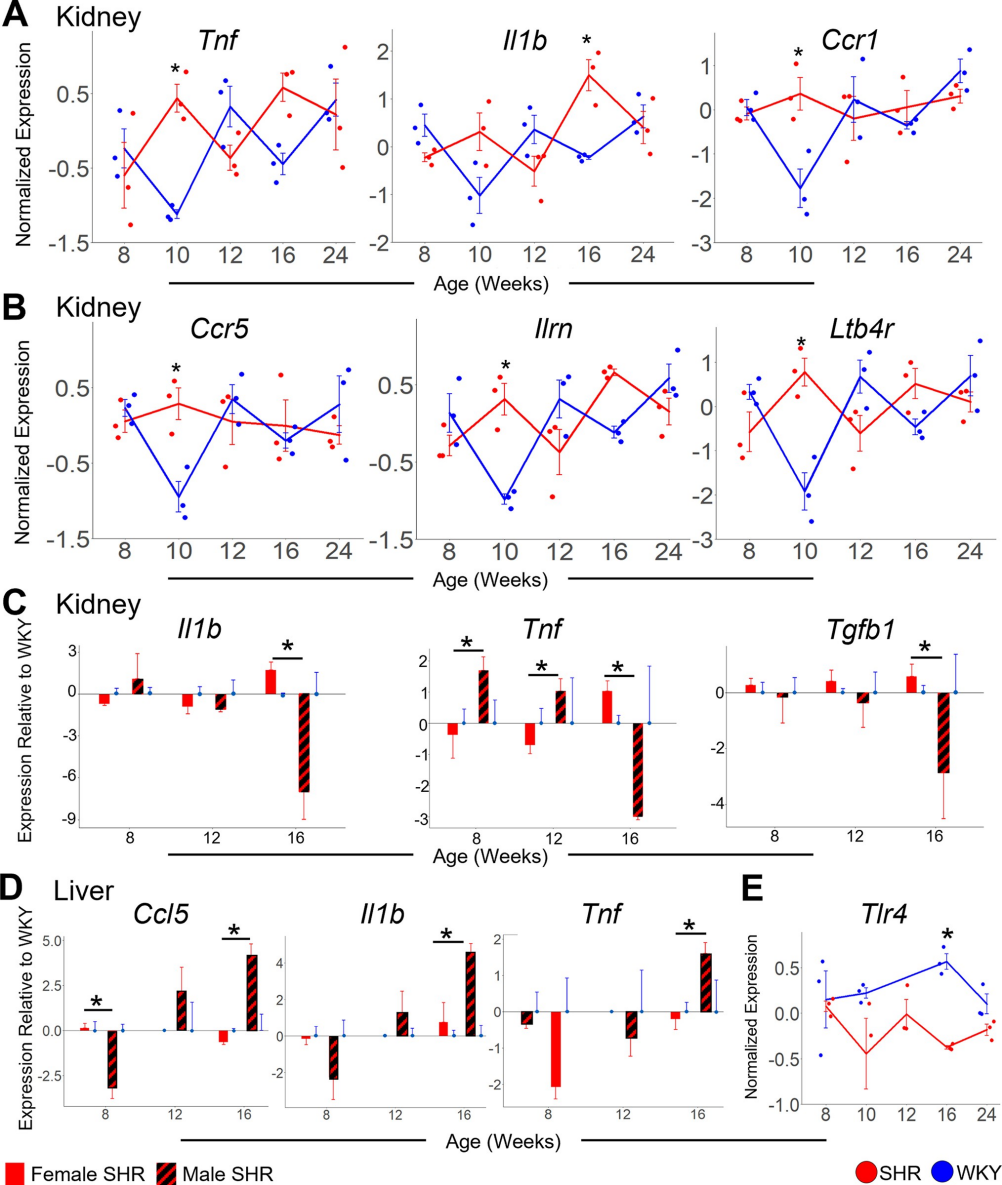
Multi-organ differential gene expression changes statistically significant at hypertension onset. A-B: Relative expression (–ΔΔCt) of *Tnf* (panel A, left), Il1b (panel A, middle), and *Ccr1* (panel A, right) in kidney as well as of *Ccr5* (panel B, left), *Ilrn* (panel B, middle), and *Ltb4r* (panel B, right) in kidney. *Tnf* is significantly upregulated in SHR at 10 weeks (p = 0.02). *Il1b* is significantly upregulated in SHR at 16 weeks (p = 0.01). *Ccr1* is significantly upregulated in SHR at 10 weeks (p = 0.006). *Ccr5* is significantly upregulated in SHR at 10 weeks (p = 0.03). *Ilrn* is significantly upregulated in SHR at 10 weeks (p = 0.002). *Ltb4r* is significantly upregulated in SHR at 10 weeks (p = 8 x 10^−4^). C: *Il1b* (left), *Tnf* (middle), and *Tgfb1* (right) expression relative to WKY in female (solid) and male (striped) SHR kidney. *Il1b* is significantly downregulated in male SHR kidney compared to female at 16 weeks (p = 6 x 10^−5^). *Tnf* is significantly upregulated in male SHR kidney compared to female at 8 (p = 7 x 10^−4^) and 12 (p = 0.006) weeks, but downregulated at 16 weeks (p = 6 x 10^−6^). *Tgfb1* is significantly decreased in male SHR kidney compared to female at 16 weeks of age (p = 0.004). D: *Ccl5* (left), *Il1b* (middle), and *Tnf* (right) expression relative to WKY in female (solid) and male (striped) SHR. *Ccl5* is significantly downregulated at 8 weeks in male SHR liver compared to female (p = 0.005). *Ccl5* is significantly upregulated at 16 weeks (p = 7 x 10^−5^) weeks in male SHR liver compared to female. *Il1b* is significantly upregulated at 16 weeks (p = 0.007) weeks in male SHR liver compared to female. *Tnf* is significantly upregulated at 16 weeks (p = 0.001) weeks in male SHR liver compared to female E: Relative expression (–ΔΔCt) of *Tlr4* in liver. *Tlr4* is significantly downregulated in SHR liver at 16 weeks compared to WKY (p = 0.046). Error Bars represent +/- standard error of the mean.

During pre-hypertension at 8 weeks of age, the sex-specific immune response is apparent at the organ level of gene expression. In female SHR, adrenal *Tlr3* (p = 0.04) and *Bcl6* (p = 2 x 10^−3^), as well as *Hmgb1* (p = 2 x 10^−3^) and *Rhoa* (p = 0.04) from the liver, are upregulated relative to WKY control (Fig 9B, 9C). Adrenal *Il1b* is downregulated in pre-hypertensive SHR vs. WKY male at 8 weeks of age [13]. These contrasting results further highlight the sex-specific role of inflammation in the adrenal gland and liver early in the development of female hypertension. In kidney, several inflammatory genes showed a fluctuating pattern of expression changes over time (Fig 10A, 10B). However, during hypertension onset at 10 weeks and robust hypertension at 16 weeks, the inflammatory markers *Tnf*, *Il1b*, *Ccr1*, *Ccr5*, *Ilrn*, and *Ltb4r* are statistically significantly upregulated in the kidney of female SHR vs WKY control (Fig 10A–10B). This analysis suggests that inflammation in the kidney may follow the earlier immune response in the adrenal gland and liver in female SHR. In male SHR vs. WKY kidney, however, some of these inflammatory markers were downregulated at 16 weeks of age, including *Il1b*, *Tnf*, and *Tgfb1* (Fig 10C). Instead, the inflammatory markers *Ccl5*, *Il1b*, and *Tnf* are upregulated at 16 weeks in the liver of male SHR vs. WKY (Fig 10D). Despite the upregulation of inflammatory markers at 16 weeks in the kidney of female SHR, *Tlr4* is instead downregulated at 16 weeks in female SHR liver (Fig 10E). The sex-specific transcriptomic trajectories of inflammatory markers at 16 weeks suggests the immune response in the kidney is female-specific, while inflammation in the liver is male-specific, during robust hypertension.

## Discussion

We identified hypertension stage-specific gene expression perturbations spanning multiple pathways and multiple organs that could explain the change in blood pressure physiology during the onset and progression of hypertension. Our experimental design prioritized higher dimensionality along multiple organs and time points, and we limited the number of replicates to n = 3 for practical purposes, to yield a rich data set on 92 genes in 146 samples to drive our multivariate statistics and network modeling. The high-throughput time series gene expression data from SHR and WKY animals of both sexes derived from visceral organs as well as multiple brainstem autonomic control regions were used to generate a data-driven dynamic network model to infer gene regulatory influences within and across organs. Our integrated molecular profiling and network modeling identified a multi-organ cascade of gene regulation underlying hypertension development. Differences in gene regulatory influences from autonomic control centers of the brainstem is a sex-dependent etiology of autonomic dysfunction in SHR. Our results suggest a diminished influence of gene regulatory control from the brainstem (specifically, the CVLM) in female SHR, but the opposite in male SHR. In female SHR, the influence of gene regulatory interactions from the adrenal gland on multiple organs, especially the spleen, is heightened. In male SHR, however, gene regulatory influence from the adrenal gland is instead weakened, while gene regulatory influence from the brainstem, particularly on the kidney, is strengthened. The earliest gene expression changes in female SHR occur in catecholaminergic processes in the adrenal gland, prior to differential expression of inflammation-related genes in the kidney. In male SHR, however, the earliest gene expression changes occur in catecholaminergic processes in the brainstem and kidney, and this is followed by an upregulation of inflammation-related gene expression in the liver and kidney. In female SHR, intra-adrenal expression of RAS-related genes is upregulated and RAS-related gene expression from the kidney-liver-lung axis is downregulated, while the opposite trends occur in male SHR.

Juvenile males have a higher prevalence of hypertension as compared to premenopausal females, but this difference disappears following menopause [42]. However, women are at a greater risk for developing hypertension-related cardiovascular disease [43].There are numerous reports of sex differences in angiotensin signaling and blood pressure [44–47], the inflammatory profile [48–52], noradrenergic content and turnover rate [52, 53], differential regulation of adrenergic receptors [54], and sympathetic activity [55] of male and female SHR. Our results support a potential mechanistic role for *Ephx2* and *Esr1* to explain the differential regulation of female-specific multi-organ effects. There have been several reports of *Ephx2* upregulation in males but not in females [40, 56, 57]. We report *Ephx2* downregulation across multiple organs and time points in female SHR. Ephx2 gene knock-out (sEH-KO) in male hypertensive mice or inhibition of EPHX2 reduced the blood pressure set point [58]. Interestingly the same treatment in female hypertensive mice showed a smaller decrement in blood pressure than in male counterparts [59–65]. Cyp450 hydrolyzes arachidonic acid into Epoxyeicosatrienoic acids (EETs), and soluble epoxide hydrolase (sEH; EPHX2) subsequently hydrolyzes EETs [63]. Such a systemic decrease in *Ephx2* is therefore expected to lead to lipid metabolic defects that may further contribute to ROS and inflammation [64].

Estrogen receptor-α (ER-α) has been shown to be expressed on T cells [65], and female ER-α−/− premenopausal mice have enhanced hypertensive outcomes compared with wild type mice, suggesting a protective role of ER-α signaling in blood pressure regulation [65]. The cardioprotective effects of female sex hormones in premenopausal women depend on ER-α [66]. ER-α activates transcription in the nucleus as well as membrane-initiated signaling, the latter of which leads to activation of endothelial NO synthase. In mice lacking either ER-α or the nuclear activating function AF2 of ER-α, Ang II increased blood pressure more than in wild type mice, suggesting that nuclear transcription is responsible for the cardioprotective effect of ER-α [66]. In our data, *Esr1* (transcript encoding ER-α) is upregulated in the adrenal gland and lung in female SHR, which may account for the lower incidence of hypertension reported previously in premenopausal females as compared to males [42]. Sex hormones have been widely reported to affect the activity of RAS components in humans, including increased plasma renin activity in men as compared to age-matched women [67]. Plasma renin activity is also increased in postmenopausal women as compared to premenopausal women [68]. In ovariectomized females, testosterone increases *Agt* mRNA levels [69] while estrogen treatment reduces *Ace* mRNA levels and activity [70]. Results from our own data corroborate these findings, indicating a downregulation of endogenous sources of systemic circulatory RAS components, including *Agt* from the liver and *Ace* from the kidney, in female SHR. All of this points to decreased systemic circulating RAS components in hypertensive females as compared to male SHR.

Our multi-organ network modeling analysis suggests a distinct shift in systemic control of hypertension from normotensive state involving a decreased influence of the CVLM and an increased influence of the adrenal gland in female SHR. CVLM neurons are sympathoinhibitory and inactivation of CVLM neurons causes hypertension via upregulated sympathetic nerve activity [71]. Our results are consistent with this autonomic functional role of CVLM, as the present data-driven network modeling predicted a lower influence of CVLM in female hypertension progression. Likewise, aldosterone production from the adrenal gland has been reported to be a female-specific manifestation of hypertension in humans and rodent models, which may be regulated by activation of intra-adrenal RAS and sex hormone receptors [72]. Our data is indicative of increased gene expression involved in intra-adrenal RAS as well as the synthesis of sex hormone receptors in the adrenal gland of female SHR. Estrogen affects central autonomic control centers, causing sympathoinhibitory effects, which may explain the lack of significant gene regulatory influence from the brainstem of female SHR [73]. In ovariectomized (OVX) rats there was an increase in sympathetic activation as well as decreased baroreflex sensitivity or vagal tone, while these effects were attenuated with estrogen treatment [74]. It was further shown that the activation of estrogen receptor 2 (ESR2) with a selective agonist in the paraventricular nucleus and rostral ventrolateral medulla of OVX rats attenuates the sympathetic nerve activity reducing blood pressure in aldosterone-induced hypertension [75]. Furthermore, estrogen treatment inhibited the development of left ventricular (LV) hypertrophy (LVH) in baroreceptor-denervated rats [76]. Together, these data highlight the inhibitory function of estrogen in the sympathetic nervous system, explaining why the gene regulatory influence of the brainstem is more prominent in male SHR but lacking in female SHR.

Sympathetic renal innervation, increased renal renin, and increased renal sodium reabsorption are directly downstream to sympathetic nervous activity [77]. Therefore, the inhibitory reports of estrogen in the sympathetic nervous system paired with our results suggesting a decreased gene regulatory influence of the brainstem in female SHR, supports our finding that the gene regulatory influence of the kidney is more prominent later in female SHR. Sympathetic nerve activity decreases with age, which supports why we see an early regulatory influence of gene expression from the brainstem as well as the kidney in male SHR. However, sympathetic nerve activity increases in the presence of weight gain and metabolic syndrome, characteristic of postmenopausal hypertensive women [77]. This may explain the increased gene regulatory activation in the kidney at later time points in female SHR. The renal sympathetic nerves have been shown to play a role in differentially regulating hypertension in young and old female SHR, with a greater decrease in blood pressure with adrenergic blockade occurring in old compared with young animals, suggesting a greater contribution of renal sympathetic nerves to hypertension development later in female animals [78]. Additionally, renal denervation was associated with reduced blood pressure in both young and old females, with a more pronounced response in old females, further supporting the relevant role of the kidney at later time points in female SHR. Chronic kidney disease has been reported to progress more slowly in females compared with men, which is consistent with our results suggesting an earlier gene regulatory influence from the kidney in male SHR vs. a later gene regulatory influence from the kidney in female SHR [79].

Our results show that kidneys of female SHR have a higher upregulation of inflammatory transcripts than male SHR at 16 weeks of age. However, blood markers for renal inflammation have been shown to be higher in males than females [80]. We speculate that lower renal inflammation previously reported in females could be partially due to anti-inflammatory mechanisms involving TGFB1. Consistent with this notion, our results show that *Tgfb1* gene expression levels steadily increase from a pre-hypertensive age of 8 weeks and peak at 16 weeks of age in the kidneys of female SHR. This may be a female-specific protective mechanism, as at 16 weeks of age *Tgfb1* levels are upregulated in the kidney of female SHR, while *Tgfb1* is downregulated in the kidney of male SHR at 16 weeks. In males, hypertension and end organ damage are T cell-dependent [81]. It has been shown that premenopausal females have mechanistically different, T cell-independent hypertension. Following adoptive transfer of male CD3+ T cells to male and female immune-deficient mice (Rag-/-) followed by Ang II infusion had different effects on both sexes. Females showed a blunted response to Ang II infusion and low levels of renal pro-inflammatory cytokines while males showed enhanced renal pro-inflammatory markers [82]. Another study of immune-deficient male mice (Rag-/-) with adoptive female CD3+ T cell transfer resulted in reduced pro-inflammatory TNF-α and interleukin-17 (IL-17) producing cells in the spleen and increases in renal anti-inflammatory interleukin-10 (IL-10) gene expression compared with when male CD3+ T cells were transferred into male Rag1−/− animals [83]. One possible explanation is the role of estrogen in regulating pro-inflammatory Th17 and Th1 cell expansion [84].

Our integrated molecular profiling and network modeling identified a stage-specific multi-organ cascade of gene regulation underlying hypertension progression. These findings suggest that the temporal organ-specific underpinnings of neurogenic hypertension development in females is mechanistically distinct from that of males. The upregulated early gene regulatory influence of adrenal gland and the downregulated role of the CLVM in female SHR is contrasted with the upregulated gene regulatory influence of the brainstem and the downregulated role of the adrenal gland in male SHR. Other differences include the gene regulatory influence of the kidney early and liver later in male SHR paired with the gene regulatory influence of liver early and kidney later in female SHR, as well as a novel gene regulatory influence of the spleen in female SHR. Finally, we report a potential mechanistic role for *Ephx2* and *Esr1* to explain the differential regulation of female-specific multi-organ effects, including vasoconstriction in the lung. It is logical to suspect that downregulation of adrenal RAS/ upregulation of systemic RAS drives hypertension onset in male SHR, while enhanced intra-adrenal RAS/ downregulated systemic RAS drives hypertension onset in females. These findings are a starting point for targeted investigations into the sex-specific etiology of hypertension. We herein present a comprehensive approach which can be adapted to study the temporal progression of a variety of chronic disease conditions, as in [85], and can leverage such results by inferring gene regulatory control networks within and across organs. Our analyses provide evidence for temporally distinct disease-specific gene activation cascades associated with dysregulated multi-organ networks.

## Materials and methods

### Animal model

All protocols were approved by the Thomas Jefferson University (TJU) Institutional Animal Care and Use Committee. Female Spontaneously Hypertensive Rat (SHR/NHsd) and Wistar Kyoto (WKY/NHsd) rat strains, corresponding to autonomic dysfunction and control phenotypes, respectively, were purchased from Envigo and used for this study. All animals were housed individually in cages to avoid animal-to-animal stress from dominance that could affect blood pressure. Animal facilities were maintained in a temperature and humidity-controlled room with 12/12 hour light cycles (lights on at Zeitgeber time = 0). Experimental procedures were carried out one week following animal arrival at our facility.

### Tissue collection

Multi-organ tissue samples were collected from SHR and WKY females at 8, 10, 12, 16, and 24 weeks of age. Rats were humanely euthanized via rapid decapitation that was preceded by 5% isoflurane in O2. We harvested the adrenal gland, kidney (cortex and medulla), liver, lung, and spleen. Tissue samples were flash frozen in RNAlater and stored at -80˚C. Fifteen animals were included in our study and 3 organ samples per strain were obtained at each time point.

### RNA extraction and BioMark RT-qPCR

RNA was extracted using Direct-Zol miniprep kit from Zymo Research. Concentration and integrity were assessed with a spectrophotometer (ND-1000 from NanoDrop, Philadelphia, PA). The reverse transcriptase reaction was performed using SuperScript VILO Master Mix (Thermo Fisher Scientific, Waltham, MA). cDNA samples were processed for 12 cycles of specific target amplification of 96 genes using TaqMan PreAmp Master Mix as per the manufacturer’s protocol (Applied Biosystems, Foster City, CA, USA) followed by real-time PCR using Evagreen intercalated dye-based approach to detect the PCR-amplified product. Intron-spanning PCR primers were designed for every assay using Primer3 [86] and BLAST [87]. Real-time PCR reactions were performed using 96x96 BioMark Dynamic Arrays (Fluidigm, South San Francisco, CA, USA) of 150 samples and 96 genes (Fig 1) enabling quantitative measurement of multiple mRNAs and samples under identical reaction conditions. Each run consisted of 30 amplification cycles (15 s at 95C, 5 s at 70C, 60 s at 60C). Two 96x96 BioMark Arrays were used to measure gene expression across the 150 samples before QC. The same serial dilution sample set was included in each chip to verify reproducibility and test for technical variability. This 6-point dilution series also serves to detect any over-amplification that may lead to a bias in the data. Samples from each animal were run across the two chips to obtain data on 96 genes per sample. Each set of chip runs contained overlapping assays that served as technical replicates to evaluate chip-to-chip variability.

### Data processing and normalization

Ct values were calculated with Fluidigm software. Individual qRT-PCR results were examined to determine the quality of the qRT-PCR based on melt-curve analysis. Samples and genes with less than 60% data were removed. Replicates between the two arrays were averaged as long as the correlation between the two were within 0.85 to 1.15.

Raw Ct values for individual samples were first separated into subsets corresponding to respective organs for subsequent normalization. Raw Ct values from each organ were normalized against the median expression level of a subset of robustly expressed genes (genes with greater than 60% working reactions) across all samples within that respective organ to obtain ΔCt values. This approach is based on previous studies for normalization that is less sensitive to outliers and not impacted by a single housekeeping gene [13, 28]. The vector of median sample expression value was chosen over potential reference genes based on comparison of stable expression across all samples against known housekeeping genes using the ‘selectHKs’ function in the NormqPCRpackage in R software for statistical analysis [88]. The following equation was used to calculate ΔCt values for each gene:

$$\Delta Ct_{gene}=Ct_{gene}‐(median\;sample\;Ct)$$ (1)

The ΔCt data were then rescaled using the median across all samples within a gene using the following equation:

$$-\Delta \Delta Ct_{gene}=‐(\Delta Ct_{gene}‐\Delta Ct_{median\_across\_samples})$$ (2)

The raw and normalized data are available online as part of a Gene Expression Omnibus (GEO) dataset (GEO reference ID: GSE227753).

### Data analysis

To compare female SHR to WKY, a two-factor ANOVA was performed using age and strain as independent and interacting factors within each organ, followed by a Tukey Honest Significant Difference (HSD) post-hoc test (p < 0.05). To obtain differential expression within a sex, WKY mean -ΔCt_gene_ were subtracted from respective SHR mean -ΔCt_gene_. For example, the mean normalized expression of three WKY kidney samples at 8 weeks of age was subtracted from the mean normalized expression of three SHR kidney samples at 8 weeks of age. These delta values were then statistically contrasted across sexes via a two-way ANOVA using age and sex as independent and interacting factors, followed by a Tukey HSD post-hoc test (p < 0.05). Sex differences were assessed between common data including 17 genes, 3 time points (8, 12, and 16 weeks), and 3 organs (adrenal gland, kidney, liver).

### Computational modeling

We used a computational modeling approach that uses Hartley Modulating Functions (HMF) to analyze the dynamics of the present data beyond the discrete time points at which samples were collected, as was applied to multi-organ time series data in male SHR and WKY in our previous study [13]. Prior to implementing the HMF method, the data were scaled to the range (0,1). One of the benefits of Hartley Modulating Functions is that they can estimate interaction coefficients that describe network dynamics and structure. The advantage of this mathematical approach is that it allows for identification of robust and accurate continuous-time models from discretely sampled data [13, 89, 90]. Instead of taking temporal derivatives, the inner products between the expression data and a set of basic functions, the Hartley Modulating Functions, are approximated using the Hartley transform to transform the data into the frequency domain, allowing for determination of the interaction coefficients k. Conceptually, the expression level of any one gene in the network model can be described as the sum of the expression profiles of all other genes multiplied by an interaction coefficient k as shown in the equation below:

$$\frac{d}{dt}E_{rg}(t)=\sum_{i}^{N_{r}}\sum_{j}^{N_{g}}k_{ij}^{\left(rg\right)}E_{ij}(t)$$ (3)

where N_r_ is the number of organs, N_g_ is the number of genes, and k_ij_^(rg)^represents the interaction term attributed to the impact gene *j* in organ *i* has on gene *g* in organ *r*.

This can be represented as:

$$\frac{d}{dt}E(t)=KE(t)$$ (4)

which is also the simplified form of the full network expressed in matrix form, where **E***(t)* represents a vector describing the expression of each gene in each organ while $\frac{d}{dt}E$ represents a vector of the corresponding derivatives. **K** represents the parameter matrix that details the influence of each gene-organ combination on every other gene-organ combination in the network. We can solve for the interaction coefficients *k*_*ij*_ by applying the HMF method which entails multiplying Eq (3) by a set of modulating functions Φ_*m*_ and integrating these products.


$$\int_{0}^{T}\Phi_{m}(\frac{d}{dt}E_{rg})dt\;=\sum_{i}^{N_{r}}\sum_{i}^{N_{g}}(\int_{0}^{T}\Phi_{m}E_{ij}dt)k_{ij}^{\left(rg\right)}$$ (5)

The modulating functions are carefully chosen such that $\frac{d}{dt}\Phi_{m}$ *(t)* = 0 at t = 0 and T = 0, where t represents the initial time point (8 weeks in our case) and T represents the final time point. These integrals can be estimated using the Hartley transform and the HMF spectral components for the expression profile and its derivative [30]. Downstream regularization techniques, known as the “elastic net” were used to avoid overfitting of the model and the optimal solutions to these equations were based on comparing the simulation results to the original data [91]. We assessed the networks in the female SHR and WKY and compared the networks between sexes.

The HMF method was employed to infer a model of continuous time course data from discrete time course data. This data were visualized as a heat map ordered for peak expression in SHR and WKY to show the cascade of gene activation patterns across organs and time points in both males and females. Interaction coefficients (*k*) between regulatory and target gene expression profiles were calculated. These relationships were filtered for interaction coefficients < -2*sd and > 2*sd from the mean. The number of organ-organ relationships over this threshold were visualized as regulatory networks specific to WKY and SHR in both males and females. Gene-gene relationships involving the adrenal gland and kidney in the top 25% and 75% were also visualized as regulatory networks specific to SHR and WKY females. Gene-gene relationships in the top 25% were visualized as regulatory networks for the subset of 17 genes in common for the male and female data.

## Supporting information

S1 Fig High throughput multi-organ multi-pathway time series gene expression data across the development of hypertension. Heat map showing normalized expression of 92 genes across 146 samples in the female SHR and WKY dataset.
(TIF)

S2 Fig Model-predicted time course of multi-organ gene expression changes. The multi-organ gene regulatory network models for male and female SHR and WKY were simulated to predict the dynamic expression of the 92 genes across the inflammatory, RAS, sympathetic, metabolism and fibrosis pathways. The simulations were performed to span the ages from 8 to 24 weeks. A: Heat map of model-predicted expression levels of 92 genes across the 24-week time course in female SHR and WKY. The genes are ordered from top to bottom according to the time point of peak expression in WKY. B: Heat map of model-predicted expression levels of 17 genes in common between male and female datasets across the 24-week time course. The genes are ordered from top to bottom according to the time point of peak expression in male SHR.
(TIF)
