# Supplementary figures and images for: Multi-organ gene expression analysis and network modeling reveal regulatory control cascades during the development of hypertension in female spontaneously hypertensive rat
Source: PLoS One. 2024 Nov 8;19(11):e0313252. doi: 10.1371/journal.pone.0313252 (PMC11548744; doi:10.1371/journal.pone.0313252)

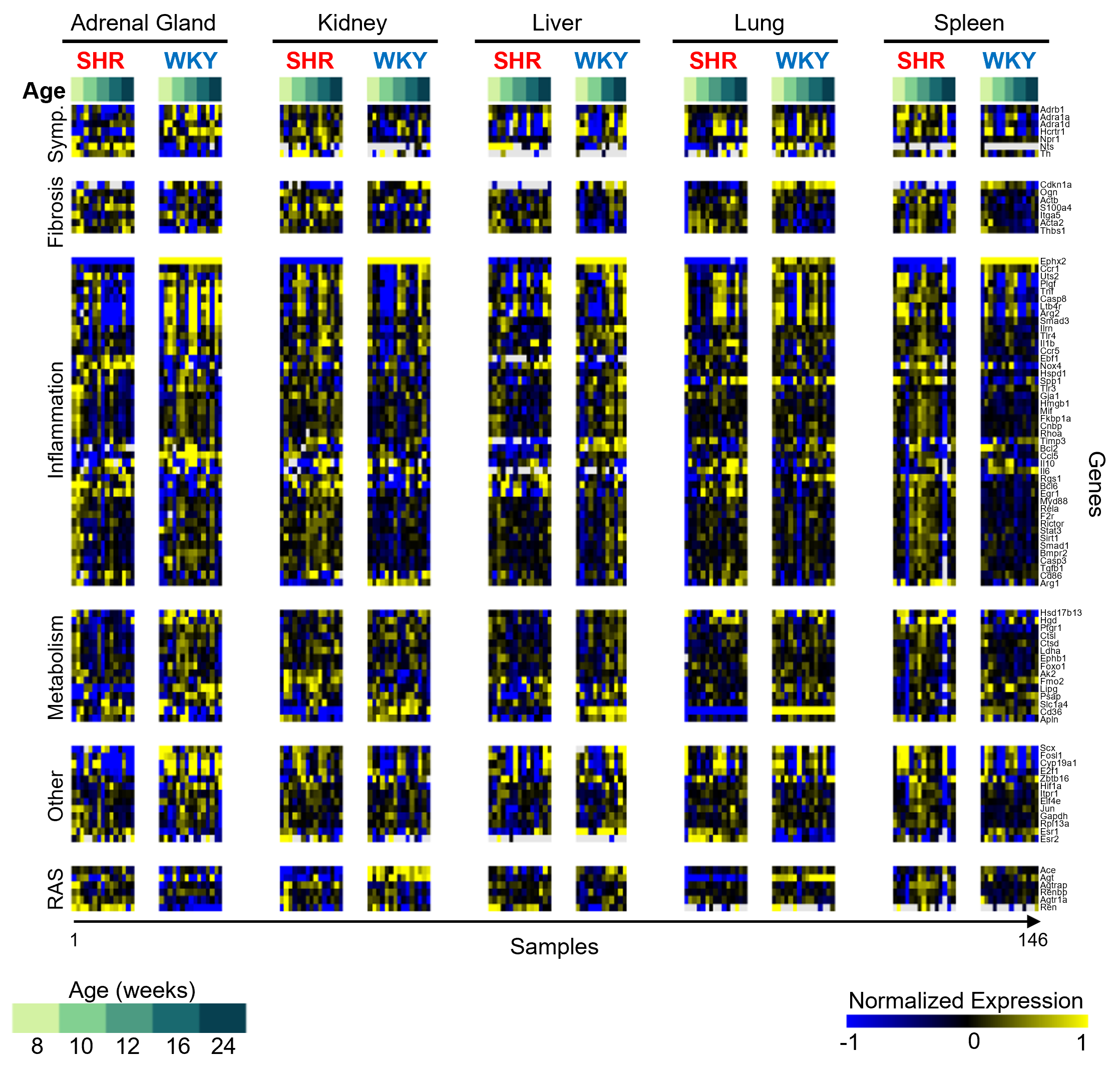

Supplement: S1 Fig — Heat map showing normalized expression of 92 genes across 146 samples in the female SHR and WKY dataset. (TIF) [file pone.0313252.s001.tif]

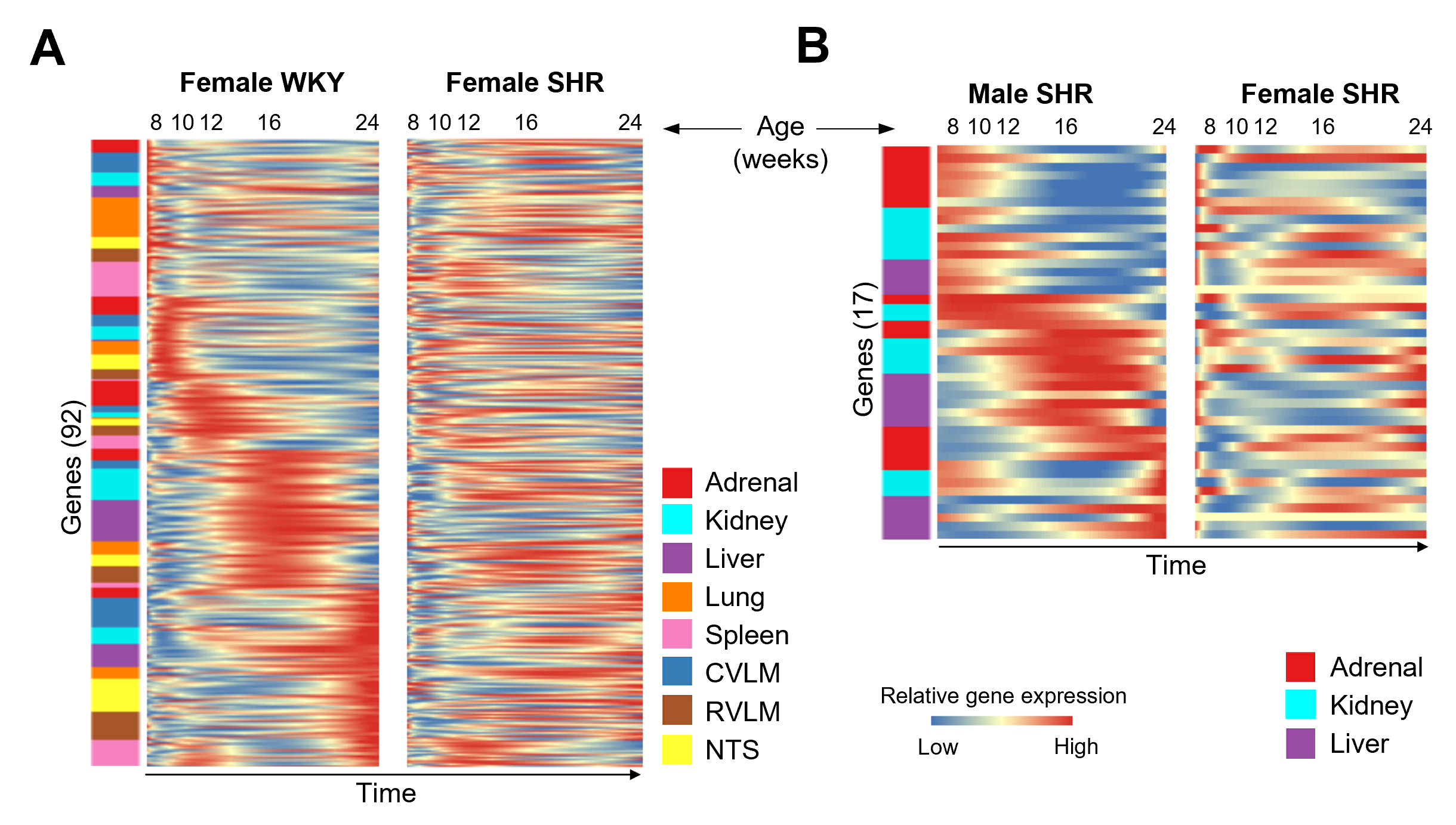

Supplement: S2 Fig — The multi-organ gene regulatory network models for male and female SHR and WKY were simulated to predict the dynamic expression of the 92 genes across the inflammatory, RAS, sympathetic, metabolism and fibrosis pathways. The simulations were performed to span the ages from 8 to 24 weeks. A: Heat map of model-predicted expression levels of 92 genes across the 24-week time course in female SHR and WKY. The genes are ordered from top to bottom according to the time point of peak expression in WKY. B: Heat map of model-predicted expression levels of 17 genes in common between male and female datasets across the 24-week time course. The genes are ordered from top to bottom according to the time point of peak expression in male SHR. (TIF) [file pone.0313252.s002.tif]
